# Effect of Microwave Intermittent Drying on the Structural and Functional Properties of Zein in Corn Kernels

**DOI:** 10.3390/foods13020207

**Published:** 2024-01-09

**Authors:** Sining Mao, Yuhan Zhou, Bin Song, Yuzhu Wu, Yu Wang, Yiran Wang, Yanjia Liu, Xiuying Xu, Chengbin Zhao, Jingsheng Liu

**Affiliations:** 1 College of Food Science and Engineering, Jilin Agricultural University, Changchun 130118, China; msn613love@163.com (S.M.); zhouyuhanhaoshuai@126.com (Y.Z.); songbinname@163.com (B.S.); wy18143044369@126.com (Y.W.); w13304475134@126.com (Y.W.); a18626941357@126.com (Y.L.); xuxiuying3288@163.com (X.X.); zhaochengbin1987@163.com (C.Z.); 2 National Engineering Research Center for Wheat and Corn Deep Processing, Changchun 130118, China

**Keywords:** microwave drying, corn kernel, zein, structural properties, functional properties

## Abstract

Microwave intermittent drying was carried out on newly harvested corn kernels to study the effects of different microwave intermittent powers (900 W, 1800 W, 2700 W, and 3600 W) on the structural and functional properties of zein in corn kernels. The results showed that microwave drying could increase the thermal stability of zein in corn kernels. The solubility, emulsification activity index, and surface hydrophobicity increased under 1800 W drying power, which was due to the unfolding of the molecular structure caused by the increase in the content of irregular structure and the decrease in the value of particle size. At a drying power of 2700 W, there was a significant increase in grain size values and β-sheet structure. This proves that at this time, the corn proteins in the kernels were subjected to the thermal effect generated by the higher microwave power, which simultaneously caused cross-linking and aggregation within the proteins to form molecular aggregates. The solubility, surface hydrophobicity, and other functional properties were reduced, while the emulsification stability was enhanced by the aggregates. The results of the study can provide a reference for the in-depth study of intermittent corn microwave drying on a wide range of applications of zein in corn kernels.

## 1. Introduction

Corn (*Zea mays* L.) is the world’s number-one food crop in terms of total production [1]. Newly harvested corn needs to be promptly reduced to a biochemically stable and safe condition (less than 14% wet basis) through drying to achieve good quality and reduce post-harvest loss rates [2]. At the present stage, hot air drying (RAD) of corn kernels has problems such as a long drying time and an unstable nutritional value of the kernels [3]. In view of the above situation, experts and scholars have conducted in-depth research on new drying technology and found that microwave drying (MWD) has the advantages of a fast drying rate and a high energy conversion rate [4]. Behera et al. [5] used RAD and MWD in paddy, and the results showed that the MWD rate was faster, rice crystallinity was lower, and energy consumption was 89.16% lower than RAD. Ma et al. [6] implemented a microwave power control strategy for potatoes by adjusting the electric field strength, which can effectively stabilize the temperature of the material, ensure the quality of the material, and prevent the drying of the potato from causing damage. However, it is important to note that many agricultural products are sensitive to heat. In the continuous drying process caused by the surface of the phenomenon of burnt paste, the quality of agricultural products is reduced. So many researchers and scholars have used intermittent microwave drying (IMD) technology for microwave drying experiments in conjunction with drying applications. Compared with continuous microwave drying, intermittent microwave drying can effectively improve the uniformity of temperature, which is conducive to reducing the possibility of quality deterioration in products. Dai et al. [7] studied the drying characteristics and quality of apple slices using the IMD technique. Xu et al. [8] used IMD technology to quantify changes in rice dehydration and improve seed cleavage rate. Kermani et al. [9] explored changes in sensory characteristics, firmness, and nutritional quality of pistachios as affected by IMD technology. These studies not only reveal that IWD has a short drying time and high product quality after drying but also indicate the feasibility of IWD applied to corn kernels.

In recent years, many studies have shown that different corn kernel drying conditions change the structural characteristics of the main components of corn, which in turn affects the nutritional quality and digestive properties of the kernels. Malumba et al. [10] explored the modification changes occurring in starch during high-temperature drying of corn kernels. The results showed that high-temperature drying would induce partial pasting of starch granules and lead to changes in physical structure, while the crystalline properties of corn starch dried at low temperatures were more pronounced. Abasi et al. [11] explored the increase in cracking rate and decrease in toughness of corn kernels due to higher internal tension at convective drying temperatures. Odjo et al. [12] showed that different fluidized bed drying conditions resulted in significant changes in the in vitro digestive characteristics of starch in corn kernels. However, there are fewer studies on the effects of changes in the drying process on the physicochemical properties of proteins in corn kernels. Zein, as the predominant storage protein in corn, accounts for about 45~50% of the total corn protein and has high biocompatibility and surface hydrophobicity [13]. Zein plays a crucial role in corn kernels, and it is of great significance to investigate the changes in the structural and functional properties of zein during the drying process in order to study the changes in the nutritional value of corn kernels. Based on the findings, microwave treatment can change the structural and functional properties of proteins. Sun et al. [14] demonstrated that microwave-treated pigeonpea flour protein disorder structure increased and solubility rose. The strength of the gel network of rice protein was found to be enhanced after the microwave treatment of rice protein by Zhong et al. [15]. Rao et al. [16] found that MWD denatured its isolated proteins during microwave treatment of millet flour, and the foaming properties were significantly reduced. At the same time, the secondary structure elements and the crystal structure were significantly changed. Therefore, we believe that microwave drying will have a greater impact on the structural and functional properties of the alcohol-soluble proteins in corn kernels, and few mechanistic studies have been reported in this area of research.

In this study, microwave intermittent drying experiments were carried out with corn kernels “Jidan 31” as the research object. It is important to optimize the efficiency of the dried product compared to the existing studies on corn kernel drying. Meanwhile, this study mainly investigates the effects of different microwave powers on the microstructure and functional properties of zein. It is of practical significance to monitor the quality of corn kernels after microwave drying, to improve the functional properties of zein, and to increase the added value of corn food products. It will provide theoretical and data support for the application of zein after microwave drying of corn kernels in related industrialization.

## 2. Materials and Methods

### 2.1. Materials and Reagents

The corn kernels were sourced from Gongzhuling City, Jilin Province, China, and had an initial moisture content of approximately 29% (±2%). Sodium dodecyl sulfate (SDS), Glycine, and 5,5′-dithiobis-2-nitrobenzoic acid (DTNB) were obtained from Shanghai Yuanye Biotechnology Co. (Shanghai, China). The remaining reagents were of analytical purity.

### 2.2. Microwave Intermittent Drying of Corn Kernels

Corn kernels without mold, impurities, or broken grains were carefully selected for testing. Test samples were weighed using a balance with an accuracy of ±0.05 g. Each batch consisted of corn kernels with a mass of 2000.0 g and an initial moisture content of 29 ± 2%. It is the initial water content of corn kernels determined according to the method of GB/T 10362-2008 [17].

**Figure 1:**
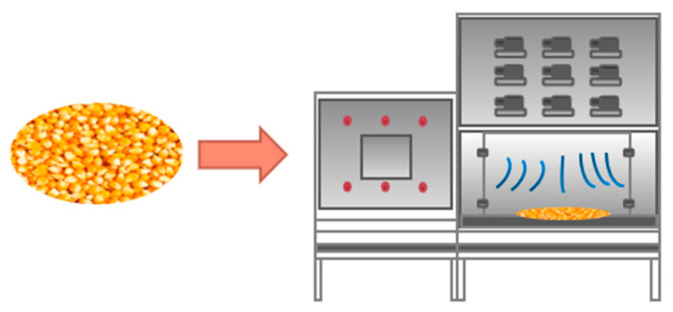
This is an image of the IMD drying test facility.

Drying using a microwave–hot air combined dryer (MDC-9-R2-9), jointly developed by Jilin Agricultural University/Shanghai Biin Mechanical and Electrical Technology Co. Intermittent microwave drying was used, and the drying power was set at 900 W, 1800 W, 2700 W, and 3600 W. The schematic diagram of the microwave intermittent drying test is shown in Figure 1. Microwave pulse ratio (PR, pulse ratio) is calculated by PR = (t_on_ + t_off_)/t_on_ [18], of which t_on_ is for the microwave on time, s, and t_off_ is for the microwave stop time, s. Corn kernels were placed in a microwave drying oven with a fixed thin layer thickness of 1.5 ± 0.1 cm and a microwave pulse ratio of 3.5 (2:5, t_on_:t_off_). The change in mass of the corn kernels before and after each set of drying was measured using a precision electronic balance. According to the national standard GB/T10463-2008 [19], the drying is stopped when the water content of the corn kernel drops to 14 ± 0.5%. A portable infrared thermometer was used to measure the temperature at three different, uniformly distributed locations in the same material layer, and the average value was taken as the average temperature of the dried corn kernels in each group.

(1) For real-time wet basis moisture content calculation for corn, the formula is as follows:

$$M_{t}=[1-\frac{G_{0}\text{×}(1-M_{0})}{G_{t}}]\text{×}100\%$$

where G_t_ was the mass of corn (g) at the moment of drying t; G_0_ was the initial mass of corn (g); and M_0_ was the initial moisture content of corn (%).

(2) For the calculation of the dry basis moisture content of corn, the formula is as follows:

$$W_{t}=\frac{100\;\times \;M_{t}}{100-\;M_{t}}$$

(3) For drying rate calculation, the formula is as follows:

$$\mathrm{DR}=\frac{W_{t_{2}}-W_{t_{1}}}{t_{2}-\;t_{1}}$$

where DR was the drying rate of corn, Wt_1_ was the dry basis moisture content of corn at the moment of t_1_ (%), and Wt_2_ was the dry basis moisture content of corn at the moment of t_2_ (%). Wt_2_ was the dry-basis water content of corn at the moment of t_2_ (%).

### 2.3. Measurement of Protein Content

The method was referred to as the Kjeldahl method for nitrogen determination as specified in GB 5009.5-2016 [20].

### 2.4. Treatments and Preparation of Zein

The method was referred to by Zhao et al. [21], with some slight modifications. After being dried, the corn kernels were pulverized into a fine powder and then sifted through an 80-mesh sieve. The resulting powder was mixed with n-hexane in a 1:3 ratio for degreasing, stirred at room temperature for 2 h, and repeated three times (each time replacing the clarified n-hexane). After that, the corn flour sample was immersed in an 80% ethanol solution with a material–liquid ratio of 1:8 and extracted at 55 °C for 3 h. After centrifugation at room temperature for 20 min at 4500 rpm, the supernatant was collected. A solution of 3% NaCl was then added to the supernatant and precipitated at 4 °C for 36 h. Following that, the precipitate was taken by centrifugation at 4500 rpm for 20 min to obtain wet zein in corn kernels. The wet zein in corn kernels was washed and centrifuged three times with deionized water. Finally, the samples were freeze-dried prior to utilization.

### 2.5. Sodium Dodecyl Sulfate Polyacrylamide Gel Electrophoresis (SDS-PAGE)

The method was referred to by Baloyi et al. [22], with some slight modifications. The electrophoresis procedure involved vertical plate gel electrophoresis, utilizing a 12% separation gel and a 5% concentration gel. The samples were prepared as a zein solution of 20 mg/mL in an 80% ethanol solution, which was then mixed with the sampling buffer containing β-mercaptoethanol according to the appropriate dilution ratio. Subsequently, the mixture was boiled for 5 min in a water bath, and then 10 μL of the solution was added to the wells of the gel plate. The voltage of the upper layer of concentrated gel was set to 80 V, while the voltage of the lower layer of separated gel was adjusted to 120 V. Subsequently, after completion of electrophoresis, the gel was carefully detached and subjected to staining with Caulmers Brilliant Blue R-250 for a duration of 30 min. Following this step, destaining was performed using an ethanol–acetic acid solution on a thermostatic oscillator until distinct bands were achieved without any background color. Finally, electrophoresis profiles were captured using the iBright CL1000.

### 2.6. Circular Binary Chromatography

The samples were diluted with an 80% ethanol solution to obtain a zein solution concentration of 0.2 mg/mL, and the measurements were conducted at a temperature of 25 °C. The determination was performed using a J-820 spectrophotometer (JASCO, Tokyo, Japan) with a scanning wavelength range set from 190 nm to 250 nm, a scanning speed of 100 nm/min, and a cuvette thickness of 2 mm. The secondary structure content of the samples was calculated using Dichro:web (available online at http://dichroweb.cryst.bbk.ac, accessed on 24 July 2023.).

### 2.7. Measurement of Free Sulfhydryl (-SH) Groups

The method was referred to by Beveridge et al. [23], with some slight modifications. The samples were prepared in an 80% ethanol solution to obtain a zein solution with a concentration of 2 mg/mL. A Tris-glycine-urea buffer (0.086 mol/L Tris, 0.09 mol/L Glycine, 0.004 mol/L EDTA, 8 mol/L Urea, pH 8.0) was prepared for subsequent experiments. The protein solution and buffer were thoroughly mixed and incubated in a water bath at 25 °C for 2 h, followed by centrifugation at 20,000 rpm at 4 °C for 20 min. Subsequently, the supernatant (4 mL) was combined with Ellman’s reagent (40 μL of a DTNB buffer with a concentration of 4 mg/mL), and the color reaction was conducted at room temperature under light protection for exactly 30 min before measuring the absorbance value at a wavelength of 412 nm using the blank control provided by the buffer solution as a reference. The calculation formula used to determine -SH group content is described below.

$$C_{SH}=\frac{73.53A_{412}D}{C_{S}}$$

where C_SH_ was the sulfhydryl content, μmol/g; 73.53 = 10^6^/13,600 (13,600 was the molar extinction coefficient of Ellman’s reagent); A_412_ nm absorbance; Cs was the mass concentration of the sample, mg/mL; and D was the dilution factor.

### 2.8. Measurement of Endogenous Fluorescence Spectra

The method was referred to by Zhang et al. [24], with some slight modifications. The samples were prepared as a 1 mg/mL zein solution, with an 80% ethanol solution used as the blank control. The excitation wavelength was set at 295 nm, while the emission wavelength ranged from 300 to 500 nm. Both the excitation and emission slit widths were fixed at 5 nm, and the voltage was maintained at 100 mV.

### 2.9. Measurement of Particle Size and Polydispersity Index

The method was referred to by Sun et al. [25], with some slight modifications. The sample was prepared as a 1 mg/mL zein solution in an 80% ethanol solution. A volume ratio of 1:1 was added to distilled water to disperse it into a 0.5 mg/mL protein–water solution, which was then shaken for 30 min to reach equilibrium. The dispersive refractive index of the sample was set at 1.450, while the refractive index of the continuous-phase water was set at 1.331. An absorbance value of 0.001 and an equilibrium time of 120 s were also established for analysis purposes using the software provided with the equipment.

### 2.10. Measurement of Surface Hydrophobicity (H_0_)

The method was referred to by Smith et al. [26], with some slight modifications. 20.00 mg of zein sample was dispersed in 2 mL of 0.01 mol/L phosphate buffer (pH 7.0) and shaken for 30 min at room temperature. Then, we added and vortexed 100 μL of a bromophenol blue indicator solution (prepared with deionized water) at a concentration of 1 mg/mL for an additional 15 min at room temperature. The mixture was centrifuged at 6000 rpm for 15 min, and the supernatant was taken and diluted tenfold in phosphate buffer. The blank control was prepared using the tenfold diluted bromophenol blue indicator solution (phosphate buffer dilution). The results were calculated using the following formula:

$$\mathrm{Bromophenol}\mathrm{blue}\mathrm{binding}\mathrm{capacity}(\mu g)=\frac{200\times (A_{0}-A_{1})}{A_{0}}$$

where A_0_ was the absorbance of the blank sample and A_1_ was the absorbance of the sample.

### 2.11. Measurement of Solubility

The method was referred to by Hu et al. [27], with some slight modifications. The sample (5.00 mg) was dispersed in 5 mL of phosphate buffer (0.01 mol/L, pH 7.0), followed by shaking at room temperature for 30 min and centrifugation at 10,000 rpm for 15 min. The resulting supernatant was utilized to determine the absorbance at 562 nm using the bicinchoninic acid (BCA) protein assay, and a standard curve was constructed with bovine serum proteins as standards. The solubility (i.e., nitrogen solubility index) was calculated according to the formula:

$$NSI=\frac{m_{1}}{m}\times 100\%$$

where *m*_1_ was the soluble protein content of the supernatant, g; *m* was the protein content in the sample, g.

### 2.12. Thermal Stability (DSC) Analysis

The method was referred to by Paraman et al. [28], with some slight modifications. The measurements were conducted using a Q-2000 Differential Scanning Calorimeter (Discovery SDT650, TA Instruments, New Castel, DE, USA). The zein sample was accurately weighed and sealed in a crucible, which was then placed on the sample holder of the DSC instrument, and the sealed empty crucible was used as a control. Temperature measurements were taken within the range of 30~180 °C at a ramp rate of 10 °C/min. Data analysis and calculations were performed using the software provided by the DSC instrument. The sample crucible was placed on the sample supporter of the DSC instrument, and the sealed empty crucible was used as a control.

### 2.13. Measurement of the Emulsification and Emulsion Stability

The method was referred to by Gao et al. [29], with some slight modifications. The zein sample was prepared by dissolving it in an 80% ethanol solution to obtain a protein concentration of 1 mg/mL. Subsequently, 12 mL of the prepared solution was added to 4 mL of soybean oil and homogenized using a homogenizer at a speed of 10,000 rpm for 2 min. A total of 100 μL of the resulting emulsion was rapidly extracted from the bottom of the test tube and added to a solution containing 5 mL of SDS (0.1%). The absorbance value at 500 nm was measured and recorded as *A*_0_. After a duration of 20 min, another aliquot consisting of 100 μL liquid from the same tube’s bottom was collected and mixed with a solution containing 5 mL SDS (0.1%). Once again, the absorbance value at 500 nm was measured and recorded as *A*_10_. The formula used for the calculation is presented below.

(1) $$EAI(m^{2}/g)=\frac{2\times 2.303}{c\times (1-\varphi )\times 10^{4}}\times A_{0}\times D$$

(2) $$ESI=\frac{A_{10}}{A_{0}}\times 100$$

where C is protein concentration, (g/mL) is soybean oil percentage (0.25), and *D* is the dilution factor.

### 2.14. Statistical Analysis

The experiments were repeated three times, and the data were processed using SPSS Statistics 21.0 software (SPSS Inc., Chicago, IL, USA). Origin 2021 software (OriginLab Corporation, Northampton, MA, USA) was used for plotting, and Duncan’s method was employed to analyze the significance of differences, with *p* < 0.05 considered significant.

## 3. Results and Discussion

### 3.1. Effect of Microwave Power on Drying Characteristics of Corn Kernels

**Figure 2:**
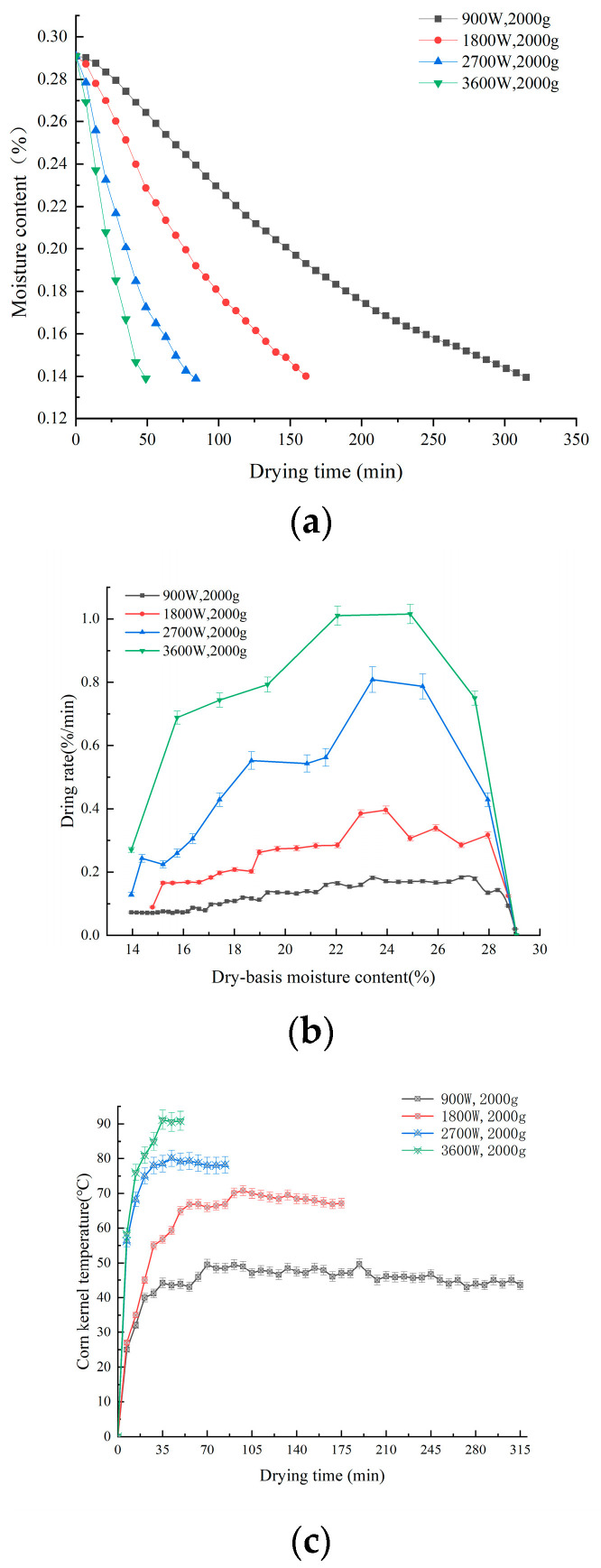
Drying curves of corn kernels under different IMD powers (**a**); drying rate curves under different IMD powers (**b**); and different temperatures of corn kernels under different IMD powers (**c**).

The use of intermittent microwave drying can balance the internal and external temperature of the material so as to reduce thermal stress and other quality losses [30]. The water loss curves of corn kernels under different microwave powers are shown in Figure 2a. The higher the microwave drying power, the faster the water loss rate of corn kernels, and the shorter the time required to reduce to safe moisture. As shown in Figure 2b,c, the variation in corn drying rate and kernel temperature is positively correlated with the microwave power. Throughout the drying phase, the drying process consists of a drying acceleration period, a constant velocity period, and a deceleration period. The kernel temperature was generally divided into rising and stabilizing periods, and the corn drying rate and kernel temperature increased with increasing microwave power. At 3600 W, the corn drying rate and kernel temperature are at their maximum. This is due to the fact that the higher the microwave power, the more microwave energy is absorbed by the corn, and the faster the polar water molecules collide inside the corn. This makes the high internal temperature increase the moisture gradient and accelerate internal heat and mass transfer [31]. Ma et al. [6] found that drying at different microwave powers resulted in changes in the nutritional quality of potatoes. Therefore, it is predicted that microwave drying power is an important factor in the effect of corn kernel precipitation rate and kernel temperature. Microwave power caused by the relevant change in corn kernel drying characteristics will have a greater impact on the quality after drying.

### 3.2. Changes in Total Protein Content and Extraction Rate of Zein from Corn Kernels

The impact of microwave power on the total protein content of corn kernels can be observed in Table 1. Initially, an increase in power led to an increase in total protein content, followed by a decrease. However, at microwave powers of 900 W and 1800 W, the change in total protein content was not significant. When the microwave power exceeded 2700 W, there was a gradual decline in total protein content. This could possibly be attributed to high-power microwaves causing internal heat aggregation and subsequent temperature rises. High temperatures cause changes in the internal protein structure, leading to protein denaturation. These findings align with Spínola et al.’s research [32], which demonstrated that high-power microwave pretreatment reduced protein content in *Arthrospira platensis*. Meanwhile, it was found that microwave power had minimal influence on the extraction rate of zein.

### 3.3. Sodium Dodecyl Sulphate–Polyacrylamide Gel Electrophoresis

**Figure 3:**
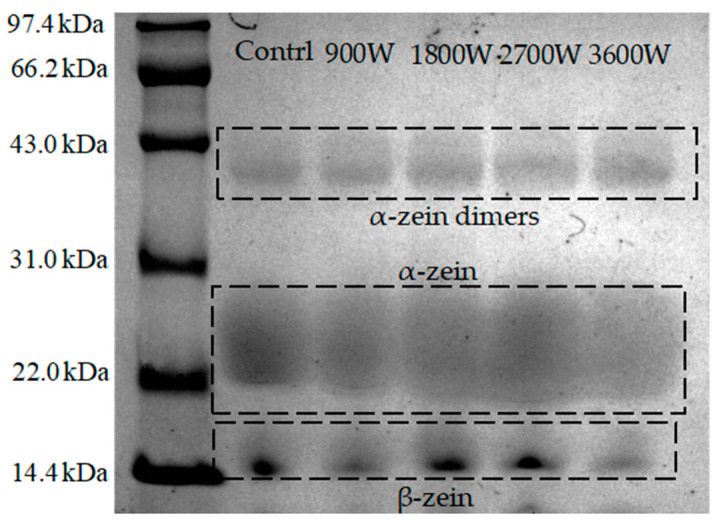
SDS-PAGE of zein under different IMD powers.

The relative molecular weight of the protein subunit can be inferred from the positioning of bands in the SDS electropherogram. Based on solubility and disulfide bond formation ability, zein was categorized into three types: α-zein, β-zein, and γ-zein, with α-zein constituting approximately 80% of the total [33]. As can be seen from Figure 3, zein in corn kernels dried with different microwave power contains the α-zein subunit (mainly 22–28 kDa) and the β-zein subunit (mainly 15 kDa). Meanwhile, a band formed by the formation of dimers by linking the cysteine residues and α-zein monomers to one another by disulfide bonding was seen in the region of 43 kDa [34]. This finding is consistent with the research conducted by Hassan et al. [35], who investigated the distribution of molecular weights in zein in corn kernels using radio-frequency heat treatment. No new bands appeared, indicating that the microwave drying process at different power levels had no influence on the composition of zein subunits in corn kernels. Further observation of the electrophoretic spectra showed that the α-zein bands tended to shift downward and then upward. The 1800 W bands are shifted downward compared to 900 W. The 1800 W bands are shifted downward. It is hypothesized that non-covalent bonds are broken, and the molecular structure unfolds and breaks down into more protein fragments. This results in a wider distribution of bands. The α-zein subunit electrophoretic bands at 2700 W were electrophoretic and deepened in color compared to other microwave drying groups. It was hypothesized that aggregation and the formation of aggregates occurred as a result of increased intermolecular interactions between the protein molecules. This is also proved by the increase in particle size values in this article. The upward shift of the bands at 3600 W was presumed to be possibly due to increased non-covalent bonding interactions and narrowing of the molecular weight distribution by oxidation reactions with disulfide bonds [36]. With the increase in microwave power, the β-zein bands showed a gradual increase. The molecular weight of β-zein was slightly larger at 3600 W, and the color became darker at 1800 W and 2700 W. These changes are related to solubility effects and heat-induced denaturation of proteins to generate large polymers [29].

### 3.4. Circular Binary Chromatography

The protein secondary structure is the regular repeating conformation of a polypeptide chain, which is closely associated with the functional properties of proteins [37]. Table 2 shows that the secondary structure content of zein in corn kernels changed as the microwave power increased, indicating that microwave action can affect the secondary structure of proteins. At 900 W, the α-helixes were 31.4% and the β-sheets were 18.5%, while the random coils and β-turns were greatly reduced. Compared with the control group, the β-sheet conformation is more likely to be formed at this power. Sun et al. [14] showed that low-power microwave drying of pigeonpea similarly increased the β-sheets of pigeonpea protein. At 1800 W, the β-sheets decreased substantially to 10.7%, and the random coils and β-turns increased to 26.4% and 29.7%, respectively. It may be due to the fact that some of the non-covalent bonds in the protein were broken and new hydrophilic sites were formed. It made the random coils increase and the molecule more prone to free curling. At this power, there will be a positive effect on solubility [38]. At 2700 W and 3600 W, the content of α-helixes decreased significantly. It is presumed that the higher radiation energy produced by high microwave power makes the molecules of zein in corn kernels appear as a non-helical phenomenon [39]. Meanwhile, their β-sheets were higher than 1800 W. Gao et al. [40] reported that the increase in the content of β-sheets can laterally reflect the occurrence of a greater degree of protein aggregation, presumably because of the cross-linking aggregation between protein molecules at 2700 W and 3600 W.

### 3.5. Free Sulfhydryl (-SH) Groups

**Figure 4:**
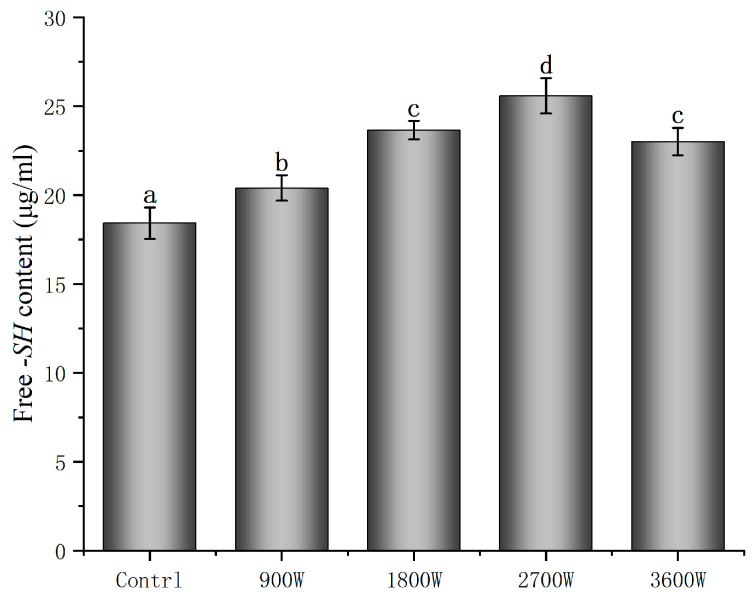
Free -SH content of zein under different IMD powers. The presence of distinct letters denotes statistically significant disparities between the samples (*p* < 0.05).

The free -SH group content of proteins occupies an important role in the structure of zein, and changes in its content can affect the functional properties of proteins [41]. As can be seen from Figure 4, under microwave intermittent drying, the protein-free sulfhydryl content was overall higher than that of the control group. Wang X [42] showed that certain microwave actions cause protein polarization and the exposure of nonpolar groups. The disulfide bond, which was not available for cross-linking, was turned into a disulfide bond with a natural-like conformation, which cross-links with free sulfhydryl groups to form a stable structure. The content of free sulfhydryl groups at 900 W was only 20.4 μmol/g, which was presumably caused by the cross-linking and folding of the molecular structure due to electromagnetic radiation, as in Wang’s study. The free -SH group content was increased to 23.66 μmol/g and 25.59 μmol/g at power levels of 1800 W and 2700 W, respectively. The increase in microwave power caused an increase in the frequency of the friction of intermolecular movement, which increased the temperature of the material. The protein conformation unfolds, making the free -SH groups originally embedded in the molecule more easily exposed to the protein surface [43]. In addition, the increase in free -SH group content may also be due to the breakage of disulfide bonds affected by microwave action to form new free sulfhydryl groups [44]. This is consistent with the results of Wang et al. [43], which showed that the free sulfhydryl content of sprouted buckwheat proteins was significantly increased by elevated microwave power and increased action time. However, the free -SH group content decreased slightly to 23.01 μmol/g at 3600 W. This may be attributed to the oxidation of some of the free -SH groups to form disulfide bonds at high temperatures, resulting in a small decrease in the free -SH group content.

### 3.6. Endogenous Fluorescence Spectra

**Figure 5:**
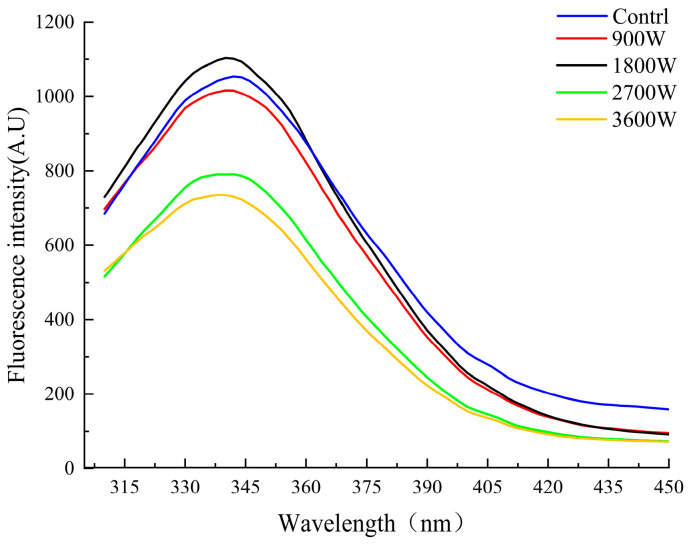
Fluorescence intensity of zein under different IMD powers.

The endogenous fluorescence spectrometry of proteins arises from the energy difference resulting from the jumps between different electronic energy levels, such as aromatic amino acid residues (tyrosine, tryptophan, and phenylalanine) [45]. Endogenous fluorescence spectrometry was used to further characterize the tertiary structure changes of proteins. As can be seen from Figure 5, the absorption wavelengths of zein in corn kernels under different microwave powers had no changes. All of them had significant changes at the absorption peak intensity of 342 nm; it showed a trend of rising first and then decreasing. The absorption peak reaches its maximum at 1800 W. This phenomenon may be due to the friction of internal polar molecules induced by the microwave electric field, which led to the unfolding of the protein conformation and an increase in the irregularly curled structure. It caused the aromatic amino acid residues originally embedded in the protein to be exposed, resulting in an increase in the intensity of the absorption peak. The intensity of the absorption peaks was significantly reduced at 2700 W and 3600 W. This phenomenon may be due to two reasons together: one was due to the protein molecules movement speed being elevated and accelerated intermolecular collision frequency and chance. Caused by non-covalent bonding and chromophore interactions, the chromophore was buried in the interior, and thus the absorption intensity was reduced. Secondly, it was possible that molecular oxygen was introduced under the environment of a high-power microwave electric field, and the endogenous fluorescence of the protein undergoes a fluorescence quenching effect. The tryptophan in the protein is oxidized, and the intensity of endogenous fluorescence is subsequently reduced [46].

### 3.7. Particle Size and Polydispersity Index

The particle size and polydispersity index of proteins can reflect the aggregation and depolymerization of proteins under the influence of the physical field from a macroscopic point of view, and their changes have an important influence on the emulsification and solubility of proteins. Table 3 reflects the changes in the particle size and polydispersity index (PDI) of zein in corn kernels under different IMD drying conditions. It was evident that the particle size and PDI value exhibited an initial decrease followed by an increase. The IMD power increased from 900 W to 1800 W, the particle size decreased from 338.3 nm to 273.27 nm, and the PDI value decreased from 15.69% to 12.14%. The smaller particle size may be attributed to the enhanced microwave electric field, which separates induced charges, exposes partially charged amino acids, causing increased electrostatic repulsion, and disrupts non-covalent bonds hindered by high electrostatic repulsion [47]. This leads to a decrease in particle size and a more dispersed state. At 2700 W and 3600 W, the particle size increased to 404.7 nm and subsequently decreased to 370.2 nm, indicating a significant increase compared to that at 1800 W. This observation can be attributed to the stretching of protein structures under high-power IMD drying conditions, leading to an increase in hydrophobic binding sites and enhanced cross-linking, thereby promoting protein aggregation [48]. Li et al. [47] also showed that as the microwave power increases, the hydrophobic interaction is enhanced and contributes to the formation of a stable mesh structure. The slight decrease in particle size at 3600 W, along with the reduction in PDI from 23.86% at 2700 W to 18.7%, was attributed to the destruction of some aggregated proteins caused by the superheated high-temperature effect. This is in agreement with the findings of Sponton et al. [49]. The SDS-PAGE electropherograms in this paper likewise validate this conclusion.

### 3.8. Surface Hydrophobicity (H_0_)

**Figure 6:**
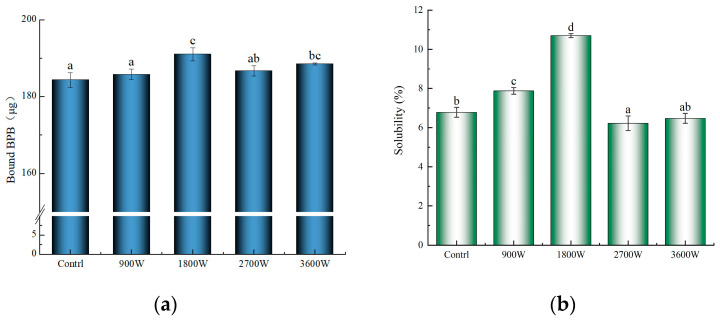
The surface hydrophobicity of zein under different IMD powers (**a**); the solubility of zein under different IMD powers (**b**). The presence of distinct letters denotes statistically significant disparities between the samples (*p* < 0.05).

The surface hydrophobicity of proteins is an important characteristic that reflects the variation in hydrophobic groups on their surface structure [50]. The bromophenol blue binding method is more applicable for assessing the surface hydrophobicity of alcohol-soluble proteins, and a higher degree of protein–bromophenol blue interaction indicates a stronger force and greater surface hydrophobicity [32]. As can be seen from Figure 6a, the surface hydrophobicity of the IMD-dried products was higher than that of the control group. Compared with the control group, the temperature of the drying system at 900 W does not exceed 50 °C, so the hydrophobic groups are less exposed, and the enhancement of surface hydrophobicity is small. At 1800 W, the protein molecular structure was further unfolded, exposing a large number of hydrophobic groups to enhance the surface hydrophobicity. Corn kernels had reduced surface hydrophobicity at 2700 W compared to protein at 1800 W. It was hypothesized that this was mainly due to the high-speed movement of polar molecules generating a large amount of heat, which caused hydrophobic residues to refold under the action of non-covalent bonds to form aggregates. Some of the hydrophobic groups that had been exposed underwent oxidative reactions and were once again encapsulated within the molecule, resulting in fewer hydrophobic groups on the surface of the protein and a decrease in surface hydrophobicity [51]. The enhanced hydrophobicity of the 3600 W surface was due to the fact that high temperatures caused some of the aggregates that had formed to decompose again.

### 3.9. Solubility

The solubility of proteins is a crucial functional property that plays a pivotal role in determining and influencing various functionalities, including emulsification and emulsion stability. From Figure 6b, it could be seen that with the increase in drying IMD power, the solubility of zein in corn kernels showed a trend of rapid increase followed by a slow decrease. This is mainly caused by changes in the secondary structure of their proteins due to the effects of temperature and electric field radiation during the drying process. The solubility was only 7.87% at 900 W, but it reached the best level of 10.70% for zein in corn kernels at 1800 W. At this time, the protein molecule is stretching after the structure of the loose protein becomes more disordered. This results in the release of polar and hydrophobic groups within the molecule, leading to an increase in molecular surface charge. Consequently, the water molecules and protein molecules in the contact range increased, improving the solubility of proteins [32]. The solubility of zein decreased to about 6.50% at 2700 W and 3600 W and was not significantly higher at 3600 W than at 2700 W. This may be mainly due to the heat-induced shift of the soluble fraction to insoluble, where proteins show a decrease in solubility through intermolecular aggregation caused by hydrophobic interactions. The smaller increase is due to the high power-induced superheating effect, which causes a small degradation of the already-generated macromolecular aggregates, resulting in a small increase in solubility. This is in agreement with Wang et al. [42], who reported that the solubility of egg white proteins also showed a first increase and then a decrease with increasing IMD power and time.

According to the conclusion of 3.8, it is concluded that the surface hydrophobicity is also improved at 1800 W. However, there is no necessary connection between solubility and surface hydrophobicity. The solubility is mainly related to the hydrophilic group, while the surface hydrophobic group is related to the hydrophobic binding site, and the molecular structure is fully unfolded at 1800 W, which causes the hydrophilic and hydrophobic groups to be exposed at the same time, so both of them are enhanced. After the analysis of particle size and SDS-PAGE at 2700 W in this paper, it is concluded that 2700 W produces aggregates so that the solubility and surface hydrophobicity are decreased.

### 3.10. Thermal Stability (DSC) 

**Figure 7:**
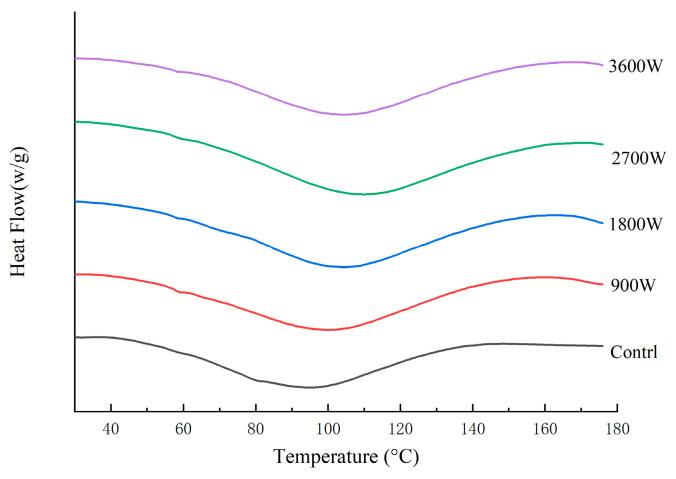
DSC thermodynamic parameters of zein under different IMD powers.

The peak protein temperature (Tp) is used to indicate the protein denaturation temperature, which can reflect the thermal stability of proteins, with higher values representing better protein stability [52]. As can be seen in Figure 7 and Table 4, the Tp of corn kernels dried with different IMD powers increased to varying degrees compared to the control group, indicating that IMD drying enhances the thermal properties of zein in corn kernels. With increasing drying power, the Tp rose from 101.36 °C at 900 W to 104.56 °C at 1800 W and peaked at 108.15 °C at 2700 W. The rise in drying power led to an increase in the Tp of proteins, possibly due to the thermal and electric field effects induced by high IMD power causing cross-linking and polymerization of protein molecules. This resulted in improved thermal properties, consistent with previous reports indicating enhanced thermal properties of globular proteins after heat treatment [53]. As the IMD drying power increased, the Tp increased from 101.36 °C at 900 W to 104.56 °C at 1800 W and reached a maximum of 108.15 °C at 2700 W. This may be due to the thermal and electric field effects generated by high IMD power, which cause cross-linking and polymerization of protein molecules, resulting in enhanced thermal properties. The Tp at 3600 W is further reduced due to the overheating effect caused by the ultra-high power. The enthalpy of denaturation (ΔH) is the value of the energy required to denature a protein by breaking hydrogen bonds (exothermic) and hydrophobic interactions (absorbing heat) [52]. The ΔH increased again to −114.1 J/g at 2700 W, presumably due to the enhancement of non-covalent bonding that maintains the molecular structure of the protein by forming a relatively stable structure. At 3600 W, the ΔH decreased to −96.7 J/g. It may be due to the fact that high heat can re-dispersed a small portion of the already-formed aggregates of denatured proteins.

### 3.11. The Emulsification and Emulsion Stability

**Figure 8:**
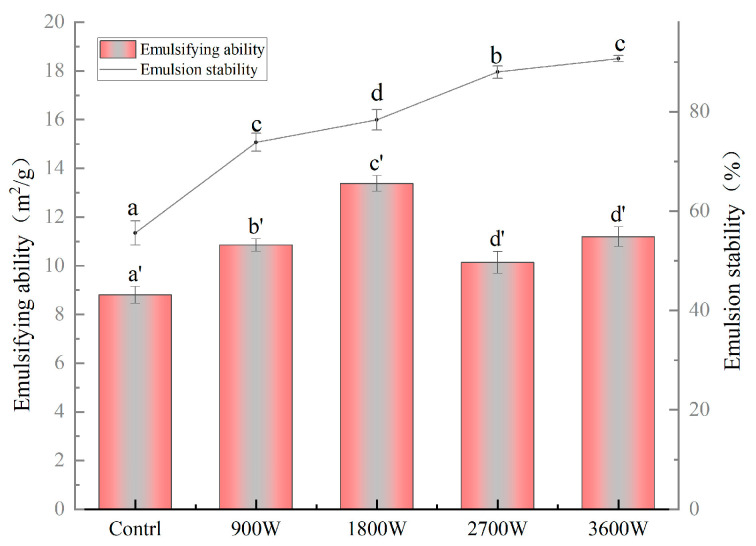
The emulsifying ability and emulsion stability of zein under different IMD powers. The presence of distinct letters denotes statistically significant disparities between the samples (*p* < 0.05).

The emulsifying properties of zein are formed mainly because it is an amphiphilic protein that can adsorb at the oil–water interface to form an oil–water film with adsorption capacity, and its emulsifying properties are related to its solubility [54]. From Figure 8, the emulsification activity and emulsion stability of zein in corn kernels under different IMD drying powers were significantly higher than those of the control group, indicating that the IMD action could improve its emulsification activity and emulsion stability. The emulsification activity of zein in corn kernels increased and then decreased, reaching its optimum at 13.8 m^2^/g when exposed to 1800 W. Based on the results of the secondary structure measurements in this paper, it is hypothesized that this was due to an increase in amphiphilicity due to a change in the protein structure from an ordered to a loosely disordered structure. It can promote the movement of more hydrophobic groups interacting with hydrophilic groups towards the oil–water interface, forming a more stable and highly elastic interfacial film adsorbed at the oil–water interface to stabilize the emulsion [50]. The emulsification activity at 2700 W and 3600 W exhibited a reduction of 27.02% and 19.32%, respectively, in comparison to that observed at 1800 W. It may be that high-speed collisions between protein molecules expose the internal hydrophobic groups and cross-link them into dense molecular aggregates with predominantly hydrophobic interactions. The overall trend in emulsification activity is consistent with the solubility of this paper. While the emulsification stability exhibited a continuous increase at 2700 W and 3600 W, it was primarily attributed to the hydrophobic group interactions, resulting in the formation of larger aggregates among protein molecules, consequently leading to enhanced emulsification stability [55].

## 4. Conclusions

In this study, the effect of microwave intermittent drying on the drying rate and temperature of corn kernels at different power levels (900 W, 1800 W, 2700 W, and 3600 W) was investigated, as well as the changing structural and functional properties of zein in corn kernels. IMD at different powers had significant effects on the drying rate and temperature of corn kernels. The structure of zein in corn kernels also changed significantly. It is mainly characterized by changes in protein secondary structure, particle size, tertiary structure, etc. At the same time, these structural changes cause alterations in the functional properties of zein in corn kernels. In particular, at 1800 W, the increase in disordered structure was able to improve the solubility, emulsification activity, and surface hydrophobicity of zein in corn kernels. At 2700 W, the thermal and emulsification stability of the zein increased significantly. In addition, drying can be further explored for the functional properties of zein in corn kernels, such as in vitro digestibility and film-forming properties, to provide theoretical support for its subsequent research.

## Figures and Tables

**Table 1:** Total protein content and extraction rate of zein in corn kernels under different IMD powers.

Sample	Total Protein Content (%)	Extraction Rate of Zein (%)
Control	8.964 ± 0.123 ^c^	4.033 ± 0.08 ^b^
900 W	8.792 ± 0.141 ^abc^	3.942 ± 0.05 ^ab^
1800 W	8.806 ± 0.101 ^bc^	3.974 ± 0.04 ^ab^
2700 W	8.596 ± 0.115 ^ab^	3.915 ± 0.04 ^a^
3600 W	8.565 ± 0.126 ^a^	3.902 ± 0.02 ^a^

The presence of distinct letters denotes statistically significant disparities between the samples (*p* < 0.05).

**Table 2:** The secondary structure of zein under different IMD powers.

Sample	α-Helix (%)	β-Sheet (%)	β-Turn (%)	Random Coil (%)
Control	31 ± 0.03 ^c^	14.7 ± 0.05 ^c^	28.5 ± 0.11 ^b^	24.8 ± 0.12 ^b^
900 W	31.4 ± 0.04 ^d^	18.5 ± 0.09 ^e^	27.1 ± 0.08 ^a^	22.9 ± 0.09 ^a^
1800 W	33.3 ± 0.02 ^e^	10.7 ± 0.02 ^a^	29.7 ± 0.06 ^c^	26.4 ± 0.10 ^e^
2700 W	29.8 ± 0.06 ^a^	15 ± 0.03 ^d^	30.1 ± 0.05 ^e^	25 ± 0.04 ^c^
3600 W	29.9 ± 0.03 ^b^	14.2 ± 0.03 ^b^	29.9 ± 0.04 ^d^	25.8 ± 0.03 ^d^

The presence of distinct letters denotes statistically significant disparities between the samples (*p* < 0.05).

**Table 3:** Particle size and PDI of zein under different IMD powers.

Sample	Particle Size (nm)	PDI (%)
Control	341.63 ±1.23 ^c^	16.46 ± 2.7 ^b^
900 W	338.30 ± 1.6 ^b^	15.69 ± 1.74 ^b^
1800 W	273.27 ± 1.92 ^a^	12.14 ± 0.79 ^a^
2700 W	404.7 ± 2.15 ^e^	23.86 ± 0.65 ^c^
3600 W	370.2 ± 2.3 ^d^	18.7 ± 1.46 ^b^

The presence of distinct letters denotes statistically significant disparities between the samples (*p* < 0.05).

**Table 4:** DSC thermodynamic parameters of zein under different IMD powers.

Sample	Tp (°C)	ΔH (J/g)
Control	96.88 ± 0.47 ^b^	−75 ± 1.25 ^a^
900 W	101.36 ± 0.35 ^d^	−108.65 ± 0.95 ^c^
1800 W	104.56 ± 0.24 ^c^	−98.67 ± 1.4 ^b^
2700 W	108.15 ± 0.57 ^e^	−114.1 ± 0.2 ^d^
3600 W	104.75 ± 0.32 ^a^	−96.7 ± 1.09 ^b^

The presence of distinct letters denotes statistically significant disparities between the samples (*p* < 0.05).

## Data Availability

Data is contained within the article.
